# Survey of viruses associated with the whitefly *Bemisia tabaci* in cucurbits in Georgia, United States

**DOI:** 10.3389/finsc.2026.1898515

**Published:** 2026-10-28

**Authors:** Mary E. Fantasia, Aasma Sharma, Sudeep Bag, Paulo S.G. Cremonez, Clint A. Penick, Kathleen M. Martin

**Affiliations:** 1 Department of Entomology and Plant Pathology, Auburn University, Auburn, AL, United States; 2 Department of Plant Pathology, University of Georgia, Tifton, GA, United States

**Keywords:** *Bemisia tabaci*, *Crinivirus*, dicistrovirus, vector pathways, virus persistence, virus-vector interactions, whiteflies

## Abstract

Whiteflies (*Bemisia tabaci*) are major agricultural pests causing over $100 million in losses to vegetable production annually through the transmission of plant viruses. Characterizing the virome of *B. tabaci* is critical for understanding vectoring potential and identifying candidate viral biocontrol agents. In this study, *B. tabaci* were collected from cucurbit crops in Tifton, Georgia, across two consecutive years, 2023 and 2024. Total RNA was extracted and ribodepleted, then sequenced with 150 bp paired-end reads on the Illumina NovaSeq 6000. Bioinformatics analyses included quality-assessing raw reads, which were trimmed prior to mapping to the *B. tabaci* reference genome (GCF_918797505.1) on the Alabama HPC, then mapping to a viral database (viral2019-03) using the Galaxy platform. After *de novo* transcriptome assembly using rnaviralSPAdes, the assembled contigs were screened for viral similarity using BLAST-based searches against the NCBI non-redundant nucleotide (nt) and non-redundant protein (nr) databases. Candidate viral contigs were evaluated by read mapping, coverage assessment, taxonomic assignment, and open reading frame prediction. The analysis identified contigs representing plant, bacteriophage, and insect-associated viruses. Near-complete cucurbit chlorotic yellows virus (CCYV) RNA1 and RNA2 sequences were assembled from both years, with higher abundance in 2024. A dicistrovirus-like sequence related to *Cripavirus* was also detected in both years and contained both major open reading frames. CCYV and the dicistrovirus-like sequence were the only viral targets with sufficient read support for genome assembly and molecular validation in both sampling years.

## Introduction

The majority of plant viruses are transmitted by insect vectors (1), yet the dynamics of these interactions remain difficult to resolve. A central challenge is distinguishing which viruses are truly associated with a given vector from those that are only transiently acquired through feeding (2). This distinction is critical for understanding how viruses persist in agricultural systems, particularly during periods when host crops are absent. Persistence may depend on vector ecology, including seasonal movement into crop fields, use of alternative host plants, or overwintering within vector populations (3–5). However, identifying vector–virus associations requires more than detection alone; it requires consistent occurrence across space and time and, where biologically appropriate, evidence of localization, circulation, or replication within vector tissues.

Whiteflies are common pests of agricultural systems, particularly *Bemisia tabaci*, which is among the most important insect vectors of plant viruses worldwide. Their broad host range and ability to move among cultivated and non-cultivated plants make them strong candidates for maintaining viral populations between cropping cycles. *Bemisia tabaci* are highly polyphagous, with more than 1,000 plant species described as hosts for the *B. tabaci* species complex (6). This broad host range allows populations to persist by shifting among crop and non-crop hosts, including “green bridge” plants that support survival and reproduction between production cycles and during winter.

In the Southeastern United States, where mild winters and diverse agricultural landscapes support year-round whitefly activity, viral transmission may be especially pronounced (7). *Bemisia tabaci* transmits viruses across multiple families, including agriculturally important begomoviruses in the family *Geminiviridae* and criniviruses in the family *Closteroviridae* (8, 9). In Georgia, USA, the 2017 outbreak underscored the scale of risk posed by whitefly–virus complexes, resulting in unprecedented economic losses of more than $200 million in cotton and vegetable crops (10–12). Viral communities associated with cucurbit production in the Southeastern United States, in particular, are highly diverse, with both aphid- and whitefly-transmitted viruses co-circulating within the same systems. For example, whitefly-transmitted viruses, such as criniviruses, have become persistent and economically significant due to environmental conditions that favor vector populations (9, 13). These viruses can also be maintained in surrounding weed species, which act as reservoirs that enable year-round persistence and reintroduction into cultivated systems, while management is further complicated by frequent mixed infections and limited availability of resistant cultivars (14, 15).

Recent advances in high-throughput sequencing have revealed that whiteflies harbor diverse viral communities, including plant viruses, insect-associated viruses, and bacteriophages. These approaches have identified widespread mixed infections of whitefly-transmitted viruses in cucurbit systems, including cucurbit chlorotic yellows virus (CCYV; genus *Crinivirus*), which was first reported in Georgia, USA, in 2021 (16). Subsequent studies have further expanded the known host range of CCYV in the region, including its detection in wild radish, supporting the hypothesis that non-crop hosts may serve as reservoirs that help maintain both the vector and virus across seasons (17). However, high-throughput sequencing also introduces a key limitation: detection does not necessarily imply biological relevance. Viruses identified in whitefly samples may reflect recent feeding events or environmental contamination rather than stable associations. As a result, there is a growing need to distinguish persistent, ecologically meaningful virus–vector relationships from transient viral signals. One approach is to assess consistency across time and space, as viruses repeatedly detected across years and locations are more likely to reflect stable associations. CCYV is transmitted semipersistently by the Mediterranean (MED; formerly Q biotype) cryptic species of the *Bemisia tabaci* complex. In MED whiteflies, development on CCYV-infected cucumber plants increased oviposition and prolonged female adult longevity (18).

The viral community associated with field-collected *B. tabaci* from Georgia was characterized across two consecutive years using RNA sequencing. The analysis evaluated whether viral detections were limited to low-abundance taxa or included viruses with sufficient read support for assembly and validation in both years. The central hypothesis was that whitefly samples would contain a mixture of plant-infecting viruses, including CCYV, and insect- or symbiont-associated viruses. Sequencing pooled whole insects from the same production region across two years allowed viral detections to be compared between sampling years and allowed identification of viral targets detected in both samples.

## Materials and methods

### Collection information

A population of at least 400 whiteflies was collected from vegetable fields in Tifton, Georgia, in fall 2023. In fall 2024, 195 whiteflies were collected from a volunteer cucumber field in Tifton, Georgia (31°26’46.0”N, 83°35’55.0”W). Whiteflies were aspirated directly from infested plants and stored in 90% ethanol until receipt in Auburn, Alabama. To standardize sampling across years, 100 individuals from each collection were selected for RNA extraction. Each 100-individual sample was transferred to 1 mL RNAlater overnight, after which the solution was removed and samples were stored at –80 °C until RNA extraction.

### RNA extraction

To identify whitefly-associated viruses, total RNA was extracted separately from two 100-whitefly samples, with one sample representing the 2023 population and one sample representing the 2024 population. Extractions were performed using the Quick-RNA Tissue/Insect Microprep Kit (Zymo Research) according to the manufacturer’s protocol. For each extraction, 100 frozen whiteflies were placed into a ZR Bashing Bead Lysis Tube containing 0.1- and 2.0-mm beads, and 800 µL RNA Lysis Buffer was added. The tube was processed in a bead beater fitted with a 2-mL tube holder. The lysate was centrifuged for 1 min to pellet debris, and the cleared supernatant was transferred to a Zymo-Spin IIICG Column in a collection tube and centrifuged. The flow-through was retained, mixed with an equal volume of 95–100% ethanol by gentle inversion, transferred to a Zymo-Spin IC Column, and centrifuged. The flow-through was discarded, 400 µL RNA Prep Buffer was added, and the column was centrifuged. The column was washed with 700 µL RNA Wash Buffer, followed by 400 µL RNA Wash Buffer, and centrifuged for 1 min to remove residual wash buffer. RNA was collected in 15 µL DNase/RNase-Free Water and stored immediately at −80 °C.

### RNA ribodepletion

Ribosomal RNA (rRNA) depletion was performed before sequencing to reduce host rRNA in total RNA samples. The Illumina Ribo-Zero Plus rRNA Depletion Kit was used according to the manufacturer’s protocol. For each sample, total RNA was incubated with Ribo-Zero Plus solution targeting cytoplasmic and mitochondrial rRNA. After hybridization and magnetic bead binding, the rRNA-depleted supernatant containing non-rRNA transcripts was collected and quantified using a NanoPhotometer NP80 (Thermo Fisher Scientific). The rRNA-depleted RNA was stored at −80 °C until library preparation.

### RNA quality assessment

RNA quality was assessed using the Agilent RNA ScreenTape system. RNA samples were thawed on ice, mixed by gentle pipetting, and diluted to 50 ng/µL with molecular-grade water. For each sample, 1 µL diluted RNA was mixed with 5 µL RNA ScreenTape sample buffer, vortexed briefly, and centrifuged. Prepared samples were loaded onto the Agilent TapeStation instrument according to the manufacturer’s instructions, and electrophoresis was performed using the standard RNA ScreenTape protocol. Electropherograms were analyzed with Agilent TapeStation software, and RNA integrity numbers (RINs) were calculated from the electropherogram profiles. Higher RIN values indicated greater RNA integrity and were used to assess sample suitability for sequencing.

### Sequencing

Ribosomal RNA-depleted RNA samples were submitted to the North Carolina State University Genomic Sciences Laboratory (Raleigh, NC, USA) for RNA library construction and sequencing. Before library construction, RNA integrity, purity, and concentration were assessed using an Agilent 4200 TapeStation with RNA ScreenTape (Agilent Technologies, USA). Messenger RNA was enriched from the rRNA-depleted RNA using oligo(dT) beads from the NEBNext Poly(A) mRNA Magnetic Isolation Module (New England Biolabs, USA). Strand-specific cDNA libraries were constructed using the NEBNext Ultra II Directional RNA Library Prep Kit and NEBNext Multiplex Oligos for Illumina (New England Biolabs) according to the manufacturer’s protocol. Briefly, mRNA was chemically fragmented and primed with random oligonucleotides for first-strand cDNA synthesis. Second-strand cDNA synthesis was performed using dUTP to preserve strand orientation. Double-stranded cDNA was purified, end-repaired, A-tailed, and ligated to sequencing adapters. Sequential AMPure XP bead selection (Beckman Coulter, USA) was used to select library fragments of 400–550 bp, including adapters. Libraries were enriched by PCR with sample-specific indices. Amplified library fragments were purified, and fragment size distribution and concentration were assessed using an Agilent 4200 TapeStation with DNA ScreenTape. Libraries were pooled in equimolar amounts and sequenced on an Illumina NovaSeq 6000 to generate 150-bp paired-end reads, yielding approximately 33 million reads per sample. Illumina Real-Time Analysis software generated raw base-call files, which were demultiplexed by sample and converted to FASTQ files for data submission.

### Bioinformatics pipeline and cross-validation

To identify viruses present in whitefly samples, bioinformatics analyses were performed using the Alabama High Performance Computing Cluster (HPC) and the Galaxy platform (19) (version 26.0.1.dev1) Raw-read quality was assessed with FastQC (20) (v0.12.1) and adapters and low-quality reads were removed with BBDuk (21) (BBMap version 38.63). A *Bemisia tabaci* reference index was built from genome assembly PGI_BMITA_v3 (NCBI RefSeq: GCF_918797505.1; GenBank: GCA_918797505.1) using Bowtie2 (22) (version 2.2.9) and host-derived reads were removed by mapping trimmed reads to this reference. Cleaned, host-filtered reads were uploaded to Galaxy for viral taxonomic classification with Kraken2 (23) (Galaxy Version 2.17.1+galaxy0 with viral2019–03 database), taxon abundance estimation with Bracken (24) (Galaxy Version 3.1+galaxy0), and assembly of classified reads with rnaviralSPAdes (25) (Galaxy Version 4.2.0+galaxy0). Resulting contigs were cross-validated by BLASTn and BLASTx (26, 27). Raw reads were mapped on the Alabama HPC with Bowtie2 (22) (version 2.2.9) and SAMtools (28) (version 1.18) to selected viral reference genomes or sequences, including Aphid lethal paralysis virus (NC_004365.1), Cucurbit chlorotic yellows virus isolate CF1–525 RNA 1 (MW629381.1), Cucurbit chlorotic yellows virus isolate CF1–525 RNA 2 (MW685456.1), Choristoneura fumiferana granulovirus (NC_008168.1), Acyrthosiphon pisum bacteriophage APSE-1 (NC_000935.1; Hamiltonella virus APSE-1), bacteriophage APSE-2 (NC_011551.1), Wuhan aphid virus 1 strain WHYC-1 segment 2 (NC_028384.1), Cotton leafroll dwarf virus (JN120901.1), Tomato spotted wilt virus isolate NC-34 clone 33 (DQ777221.1), Pepper chlorotic spot virus isolate PCSV-bidens (MG656987.1), Big Sioux River virus strain Kenya P9 (NC_035184.1), and Culex dicistrovirus 1 strain CDicV1/Fresno (MH188004.1). Read mappings were visualized with Integrative Genomics Viewer (IGV) v2.19.6 (29).

Consensus sequences were generated from read mappings on the Alabama HPC using SAMtools, BCFtools (30) (version 1.13), GATK (31) (version 4.4.0.0), and VCFtools (32) (version 0.1.16). Kraken2-unclassified assembled Kraken2-unclassified reads with SPAdes (33) (Galaxy Version 4.2.0+galaxy0). Assembled contigs from unclassified reads were screened with DIAMOND (34) (v2.0.4.142) and MEGAN (35) (v6.12.3) to identify additional viral sequences.

### Confirmation of species identification through sequence analysis

To investigate *B. tabaci* population identity, trimmed reads were assembled into contigs, and the resulting contigs were screened against a curated Aleyrodidae cytochrome c oxidase subunit I (COI/COX1) nucleotide reference database. The reference database contained more than 9,000 accessions, with sequence lengths ranging from 133 to 1,542 bp. A COI BLAST database was created from the curated Aleyrodidae COI reference panel using BLAST+ v2.15.0 on the Alabama HPC. Assembled contigs were queried against this database with BLASTn to generate a hit table. The top hit for each contig was identified, and COI-assigned contigs were extracted for downstream sequence identity analysis.

### R analysis

Bracken output tables were imported into RStudio (36) to summarize viral composition across sampling years. For each year, Bracken-estimated read counts were used to calculate the relative abundance of each viral taxon as a proportion of total Bracken-estimated virus-assigned reads for that sample. Viral taxa reported in the 2023 and 2024 Bracken outputs were compared by presence or absence to identify taxa shared between years and taxa reported in only one year. Bracken table summarization and visualization were performed in R.

### Phylogenetic analysis

Maximum likelihood phylogenetic analysis was performed to evaluate the placement of the whitefly-associated dicistrovirus-like sequence relative to aphid lethal paralysis virus (ALPV), Culex dicistrovirus 1, and other insect-associated dicistroviruses. The analysis was conducted using IQ-TREE (37) (version 2.2.6) with the following GenBank accessions for complete dicistrovirus genomes: MT195550.1, MT783302.1, MW357714.1, MZ822074.1, MZ822070.1, MZ822071.1, MZ822072.1, MZ822069.1, MZ822073.1, MH188004.1, MH188005.1, MH476200.1, MH320557.1, MH477289.1, MH459180.1, MH727530.1, MH727529.1, MH727526.1, MT176242.1, MN231041.1, AF014388.1, AF183905.1, AF218039.1, AF536531.1, AY275710.1, AY634314.1, AF150629.1, EF219380.1, KF500001.1, KX657786.1, KX588249.1, KY354239.1, and MH714707.1.

Maximum likelihood phylogenetic analyses were also performed to compare CCYV sequences recovered from the 2023 and 2024 whitefly samples with regional and publicly available CCYV isolates. Analyses were conducted using IQ-TREE (37) (version 2.2.6). The RNA1 analysis included AB523788.1, JN641883.1, JQ904628.1, KY400636.1, KY618798.1, KY400635.1, MW768903.1, OP912400.1, and OQ190463.1. The RNA2 analysis included MW685456.1, OM489401.1, MW629380.1, AB523789.1, MH819191.1, JN126046.1, KY400633.1, JN126045.1, and MW768904.1.

### Validation of viral sequences

To validate selected viral sequences, the total RNA used for sequencing was also used to synthesize cDNA with the Thermo Scientific RevertAid First Strand cDNA Synthesis Kit. The WF_DT-R4 primer (**Table 1**) was used for cDNA synthesis of the dicistrovirus target, and random hexamers were used for cucurbit chlorotic yellows virus (CCYV). cDNA synthesis followed the manufacturer’s protocol for gene-specific and random-primed reactions. Validation primers were designed in SnapGene version 8.2.2 using consensus sequences generated from read mapping to *Culex dicistrovirus 1* strain CDicV1/Fresno (MH188004.1) and CCYV isolate CF1–525 RNA segment 1 (MW629381.1). The resulting cDNA was used for PCR with the New England Biolabs Phusion High-Fidelity PCR Kit and the primers listed in **Table 1**. PCR products were separated by gel electrophoresis and imaged using the Bio-Rad Gel Doc EZ Imager.

**Table 1:** Primer sequences used for molecular validation with reverse transcriptase PCR.

Purpose	Primer name	Primer sequence	TM	Expected amplicon size (bp)
	WF_DT-F7	TGCCCGCATGTAAAGGATGT	60	
Dicistrovirus	WF_DT-R6	TGGGCGCAAATATCCAGGTT	60	995
For cDNA	WF_DT-R4	GGCACACCTATTATATGCAACG	60	
	WF_CCYV-F4	AGTGTACGAGATTCATTAAAGATGA	58	
Crinivirus	WF_CCYV-R6	CCGTCCACACATTCTGGTGA	59	953

## Results

### Sequencing

Raw sequencing output totaled 132,442,040 reads for the 2023 sample and 134,676,972 reads for the 2024 sample. FastQC analysis indicated high overall base quality, limited adapter contamination, and GC content distributions within expected ranges. Reads were retained for adapter trimming, host-read removal, and downstream viral sequence analysis.

### Bioinformatics and cross-validation

Kraken2 and Bracken analyses assigned viral reads to plant-, insect-, and bacteriophage-associated taxa in both sampling years. In 2023, viral reads were assigned to cucurbit chlorotic yellows virus (CCYV), aphid lethal paralysis virus (ALPV), tomato spotted wilt tospovirus (TSWV), pepper chlorotic spot virus (PCSV), Wuhan aphid virus 1, Hubei picorna-like virus 15, Wuhan insect virus 9, Hamiltonella virus APSE1, and bacteriophage APSE-2 (**Table 2**). In 2024, viral reads were assigned to CCYV, ALPV, Big Sioux River virus (BSRV), Hubei picorna-like virus 15, Wuhan insect virus 33, Hubei Wuhan insect virus 9, cotton leafroll dwarf virus (CLRDV), Wuhan aphid virus 1, Hamiltonella virus APSE1, and bacteriophage APSE-2 (**Table 3**). Relative abundance estimates from Bracken showed that CCYV and the ALPV/dicistrovirus-associated assignment accounted for most virus-assigned reads in both years (**Figure 1A**). Presence–absence comparison showed that several viral taxa were reported in both years, whereas others were year-specific low-abundance assignments (**Figure 1B**).

**Table 2:** Bracken report showing relative abundances of reported viral taxa for 2023.

Name	Taxonomy id	Taxonomy lvl	Kraken assigned reads	Added reads	New Est. reads	Fraction total reads
Cucurbit chlorotic yellows virus	558690	S	118	0	118	0.5
Aphid lethal paralysis virus	209529	S	98	0	98	0.41525
Tomato spotted wilt tospovirus	1933298	S	2	0	2	0.00847
Pepper chlorotic spot virus	1414655	S	1	0	1	0.00424
Wuhan aphid virus 1	1746067	S	2	0	2	0.00847
Hubei picorna-like virus 15	1923094	S	1	0	1	0.00424
Wuhan insect virus 9	1923740	S	1	0	1	0.00424
Hamiltonella virus APSE1	67571	S	7	0	7	0.02966
Bacteriophage APSE-2	340054	S	4	0	4	0.01695

**Table 3:** Bracken report showing relative abundances of reported viral taxa for 2024.

Name	Taxonomy id	Taxonomy lvl	Kraken assigned reads	Added reads	New Est. reads	Fraction total reads
Cucurbit chlorotic yellows virus	558690	S	2470	0	2470	0.82224
Aphid lethal paralysis virus	209529	S	444	0	444	0.1478
Big Sioux River virus	1041883	S	1	0	1	0.00033
Hubei picorna-like virus 15	1923094	S	20	0	20	0.00666
Wuhan insect virus 33	1923736	S	1	12	13	0.00433
Hubei Wuhan insect virus 9	1922833	S	1	1	2	0.00067
Cotton leafroll dwarf virus	312295	S	18	0	18	0.00599
Wuhan aphid virus 1	1746067	S	2	0	2	0.00067
Hamiltonella virus APSE1	67571	S	22	0	22	0.00732
Bacteriophage APSE-2	340054	S	9	0	9	0.003
Human mastadenovirus C	129951	S	2	0	2	0.00067
Choristoneura fumiferana granulovirus	56947	S	1	0	1	0.00033

**Figure 1:**
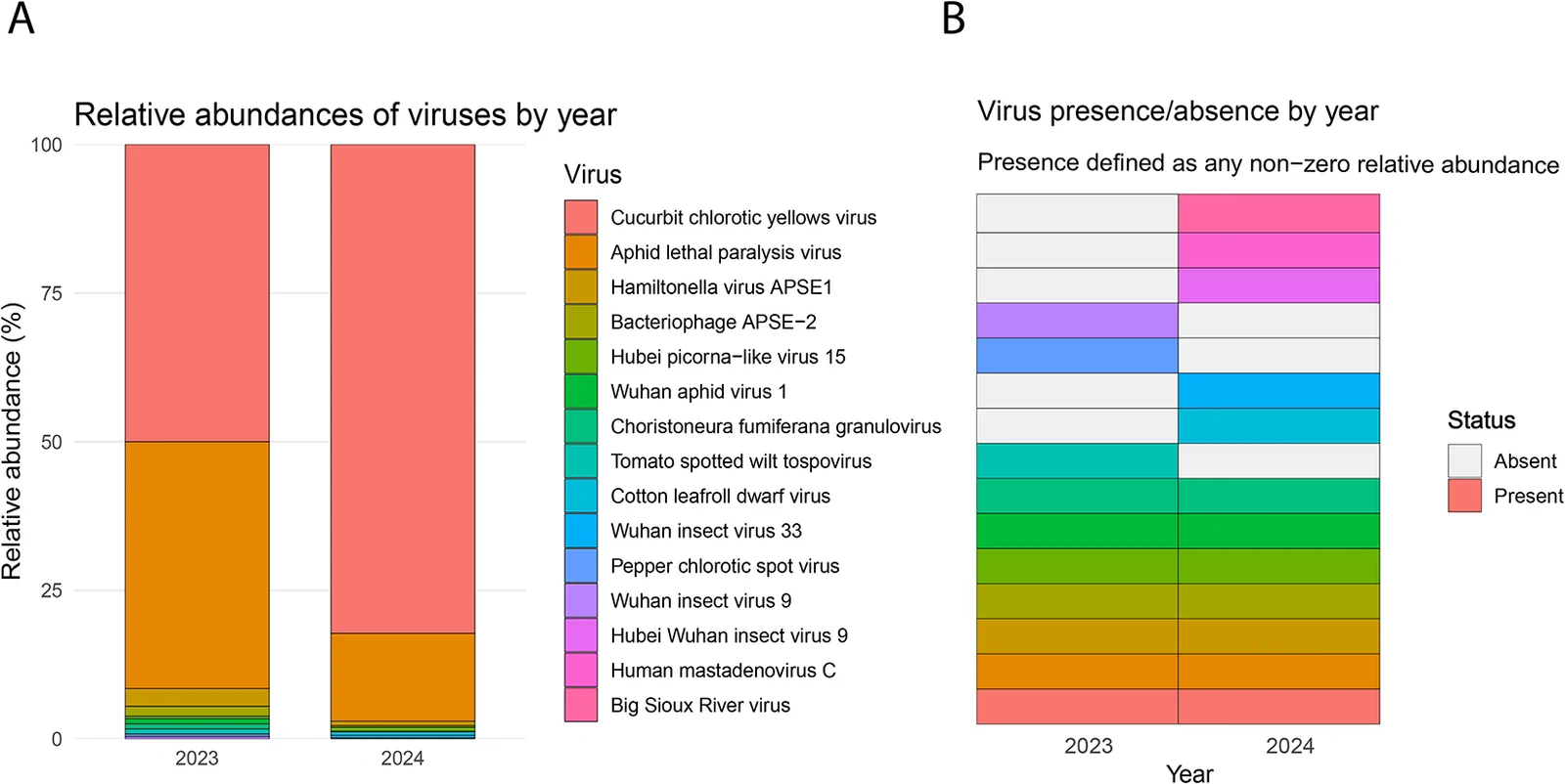
Results from analysis in R studio. Panel **(A)** shows all viruses reported by Kraken2 and their estimated relative abundances from Bracken. Panel **(B)** shows presence and absence of each virus for each year that we sampled.

Kraken2 assigned 265 reads in 2023 and 3,065 reads in 2024 to viral taxa, representing 0.0017% and 0.022% of reads, respectively. Most virus-assigned reads in both years corresponded to CCYV and an ALPV/dicistrovirus-associated assignment, whereas other plant-, insect-, and bacteriophage-associated taxa were represented by low read counts. The 2024 sample contained more virus-assigned reads and more viral taxonomic assignments than the 2023 sample. Assembly of Kraken2-classified reads produced 15 contigs in 2023 and 34 contigs in 2024, totaling 4,586 bp and 22,938 bp, respectively, with N50 values of 309 bp and 1,257 bp. Assembly of Kraken2-unclassified reads produced 12,870 contigs totaling 4,855,816 bp, with contig lengths of 78–14,876 bp and an N50 of 6,870 bp.

BLASTn and BLASTx searches supported viral assignments for the highest-coverage contigs, including a dicistrovirus-associated contig in 2023 and a cucurbit chlorotic yellows virus (CCYV) contig in 2024 (**Tables 4**, **5**). Read mapping to selected viral reference genomes showed high coverage for the dicistrovirus-associated reference and CCYV, whereas other viral taxa assigned by Kraken2 had little or no genome coverage. The dicistrovirus-associated reads mapped to the ALPV reference with 89% genome coverage in 2023 and 99% in 2024, but BLASTn identified Culex dicistrovirus 1 strain CDicV1/Fresno (MH188004.1) as the closest nucleotide match for the 2023 contig. Mapping to the Culex dicistrovirus 1 reference covered 95% of the genome in 2023 and 99% in 2024. CCYV RNA1 coverage was 59% in 2023 and 99% in 2024 (**Table 6**). MEGAN analysis did not identify additional viral taxa among Kraken2-unclassified reads.

**Table 4:** Top hits from cross-validation using BLASTn.

Year	Proposed name (accession number)	Best match (accession number)	Best match length (bp)	Query length (bp)	Alignment length (bp)
2023	Aphid lethal paralysis virus, complete genome (NC_004365.1)	Culex dicistrovirus 1 strain CDicV1/Fresno, complete genome (MH188004.1)	9811	497	490
2024	Cucurbit chlorotic yellows virus isolate AL3 RNA-dependent RNA polymerase protein (RdRp) gene, partial cds (MZ073349.1)	Cucurbit chlorotic yellows virus isolate CF1-525_1 segment 1, complete sequence (MW629381.1)	8607	6264	6191

**Table 5:** Top hits from cross-validation using BLASTx.

Year	Proposed name (accession number)	Best match (accession number)	Best match length (bp)	Alignment length (bp)	Amino acid identity (%)
2023	nonstructural polyprotein [Dicistroviridae sp.] (XOD50197.1)	nonstructural polyprotein [Aphid lethal paralysis virus] (NP_733845.1)	577	947	97.58%
2024	ORF1a polyprotein [Cucurbit chlorotic yellows virus] (QTQ95093.1)	ORF1a/1b fusion polyprotein [Cucurbit chlorotic yellows virus] (UXR26842.1)	2492	7455	99.77%

**Table 6:** Mapping statistics for viruses of interest.

Year	Virus	Total raw reads	Aligned reads	Percent alignment
2023	Aphid lethal paralysis virus	33669243	467	0.001387023
2024	Aphid lethal paralysis virus	33110510	2450	0.007399463
2023	Culex dicistrovirus 1	33669243	514	0.001526616
2024	Culex dicistrovirus 1	33110510	2771	0.008368944
2023	Curcubit chlorotic yellow virus RNA segment 1	33669243	98	0.000291067
2024	Curcubit chlorotic yellow virus RNA segment 1	33110510	1980	0.005979974
2023	Curcubit chlorotic yellow virus RNA segment 2	33669243	44	0.000130683
2024	Curcubit chlorotic yellow virus RNA segment 2	33110510	948	0.002863139

### Confirmation of species identification

COI-based sequence analysis supported a dominant MEAM1 signal in both sampling years. In 2023, one long COI contig aligned 100% to MEAM1, while additional contigs showed similarity to non-MEAM1 reference sequences, including Asia II-like sequences. In 2024, the strongest COI assignment was also MEAM1, although several shorter, lower-coverage contigs matched other *Bemisia* mitotypes. These results suggest that both samples were dominated by MEAM1, with stronger evidence for additional non-MEAM1 COI assignments in 2023 (**Table 7**).

**Table 7:** Top hits from COI reference panel and identity statistics.

Sample	Gene region approx	Contig ID	Query length (bp)	Best alignment length (bp)	Best percent identity	Best category	Best hit title	MED percent identity	MEAM1 percent identity	Asia II percent identity	Bemisia other percent identity
*WF_2023*	COI/cox1	NODE_19615_length_463_cov_1.284615_g18861_i0	463	89	91.01	SSA	Bemisia tabaci isolate CA_AD_38 clone SSA3 cytochrome c oxidase subunit I (COX1)	–	–	–	90
*WF_2023*	COI/cox1	NODE_34114_length_333_cov_1.200000_g33359_i0	333	116	93.1	Bemisia_other	Bemisia tabaci haplotype Hap4 COX1	–	–	–	93.1
*WF_2023*	COI/cox1	NODE_3620_length_1455_cov_13.651230_g3115_i0	1455	1455	100	MEAM1	MEAM1 COI	93.52	100	93.52	100
*WF_2023*	COI/cox1	NODE_610_length_3333_cov_253.642331_g516_i0	3333	1527	98.95	Asia_II	Asia II COI	98.95	93.58	98.95	99.76
*WF_2023*	COI/cox1	NODE_611_length_3333_cov_248.833436_g516_i1	3333	1527	98.95	Asia_II	Asia II COI	98.95	93.58	98.95	99.76
*WF_2024*	COI/cox1	NODE_22280_length_541_cov_1.726496_g21311_i0	541	98	93.88	Bemisia_other	VECTOR35 COI	–	–	–	93.88
*WF_2024*	COI/cox1	NODE_24402_length_510_cov_1.729977_g23432_i0	510	89	91.01	SSA	SSA3 COI	–	–	–	90
*WF_2024*	COI/cox1	NODE_38892_length_383_cov_1.712903_g37920_i0	383	83	97.59	Bemisia_other	Italy3 COI	–	–	–	97.59
*WF_2024*	COI/cox1	NODE_837_length_3686_cov_475.513147_g699_i0	3686	1537	100	MEAM1	MEAM1 COI	93.5	100	93.5	100
*WF_2024*	COI/cox1	NODE_86999_length_252_cov_0.871508_g86027_i0	252	177	91.53	Bemisia_other	JMC1 COI	–	–	–	91.53
*WF_2024*	COI/cox1	NODE_91515_length_249_cov_0.886364_g90543_i0	249	165	93.94	MEAM1	Iraq MEAM1 COI	–	93.94	–	91.34
*WF_2024*	COI/cox1	NODE_98955_length_244_cov_0.912281_g97983_i0	244	73	91.78	MEAM1	MEAM1 haplotype	–	91.78	–	–

Grammarly was used in order to improve grammar and readability. All work was again read and approved by the authors.

### Phylogenetic analysis

Phylogenetic analysis placed the whitefly-associated dicistrovirus-like sequence within a dicistrovirus clade that included aphid lethal paralysis virus (ALPV) and Culex dicistrovirus 1 strain CDicV1/Fresno. Although Kraken2 assigned reads to ALPV, BLASTn identified Culex dicistrovirus *1* strain CDicV1/Fresno as the closest nucleotide match. The sequence shared 95% amino acid identity in ORF1 and 91–92% identity in ORF2 with related dicistroviruses, supporting placement near Culex dicistrovirus 1 and ALPV but not, by itself, resolving species or strain status. In the phylogeny, the sequence clustered closest to Culex dicistrovirus 1, with ALPV positioned in the same broader dicistrovirus clade (**Figure 2**).

**Figure 2:**
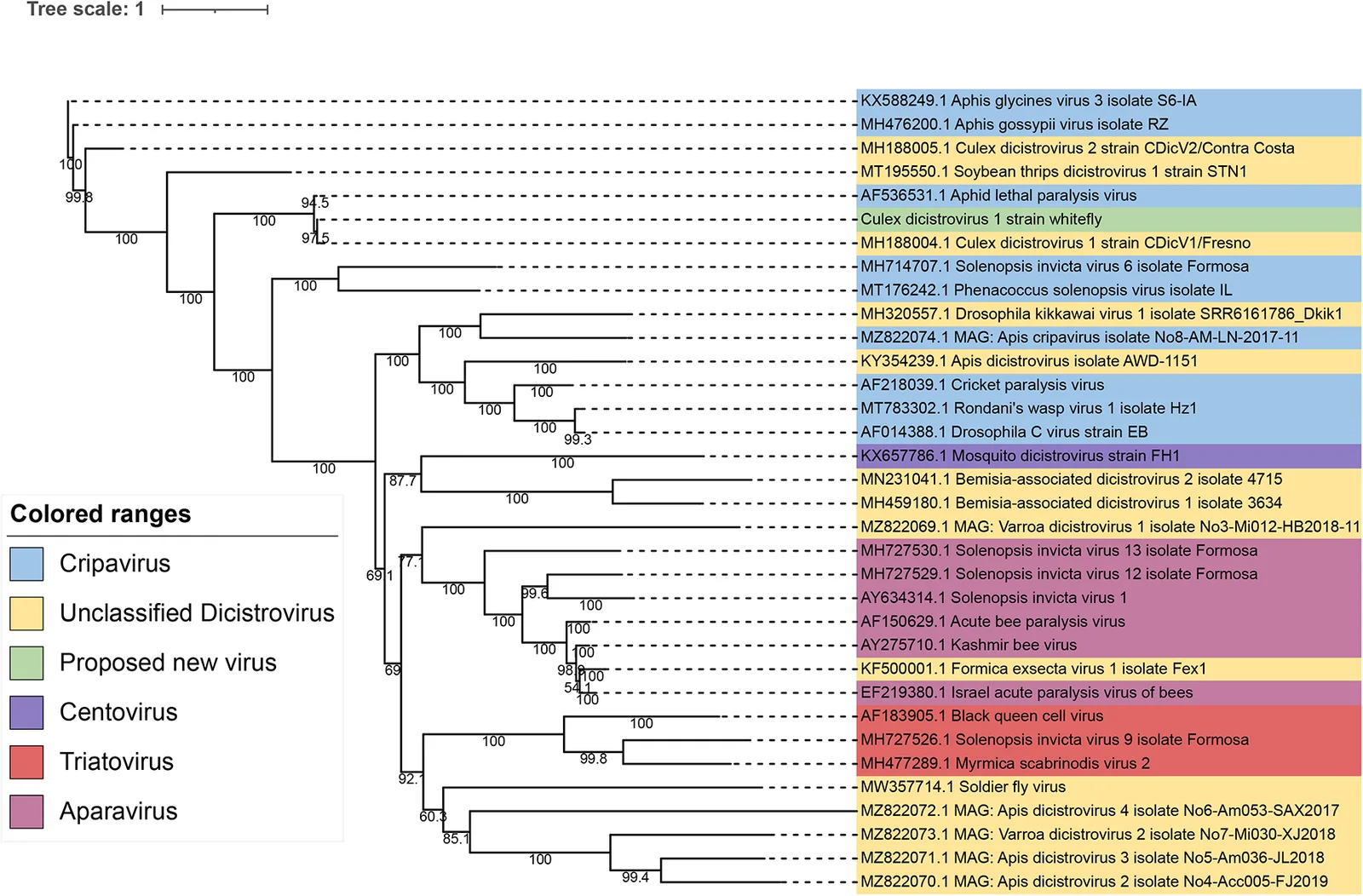
Phylogenetic tree evaluating relatedness of our proposed new virus strain to other insect-specific dicistroviruses.

Phylogenetic analyses of CCYV RNA1 and RNA2 were performed to compare sequences recovered from the 2023 and 2024 whitefly samples with previously reported U.S. isolates. Both RNA1 (**Figure 3A**) and RNA2 (**Figure 3B**) clustered with previously reported CCYV isolates from the United States and did not form a distinct clade. Lower bootstrap support in the RNA1 tree may reflect limited phylogenetic signal in the analyzed region, whereas RNA2 showed greater separation among isolates. Overall, the RNA1 and RNA2 trees supported close relatedness between CCYV sequences recovered in this study and previously reported regional isolates, rather than evidence for a distinct new strain.

**Figure 3:**
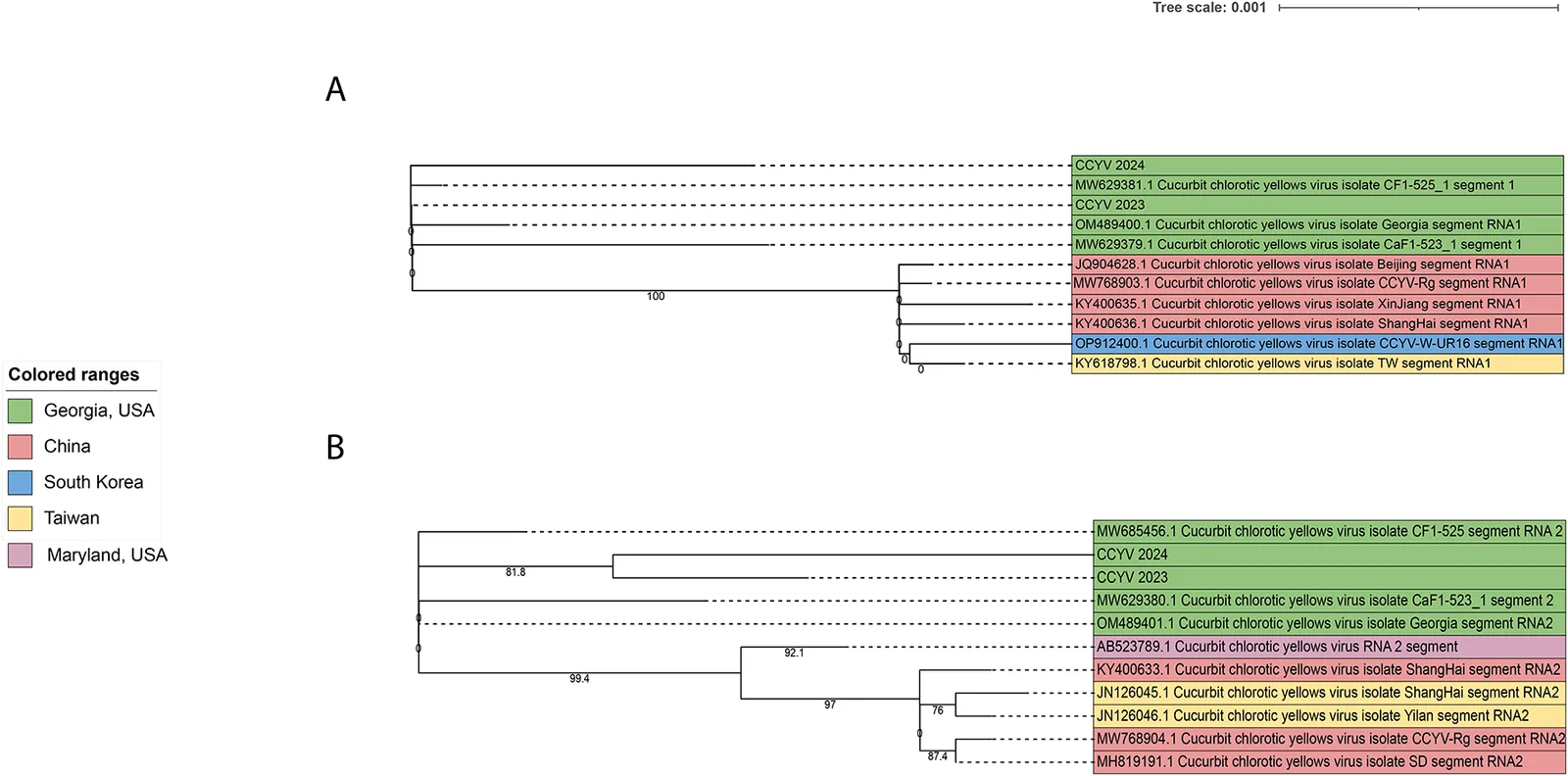
Phylogenetic tree evaluating relatedness of CCYV found in our samples to other CCYV isolates found in the US. **(A)** Shows RNA segment 1 phylogenetic analysis. **(B)** Shows RNA segment 2 phylogenetic analysis.

### Validation of viral sequences

Molecular validation focused on the two viral targets with the highest read support and genome coverage: the dicistrovirus-like sequence related to *Culex dicistrovirus 1* and CCYV. The expected 995-bp dicistrovirus amplicon was detected in both the 2023 and 2024 samples (**Figure 4A**). The expected 953-bp CCYV RNA1 amplicon was also detected in both years (**Figure 4B**).

**Figure 4:**
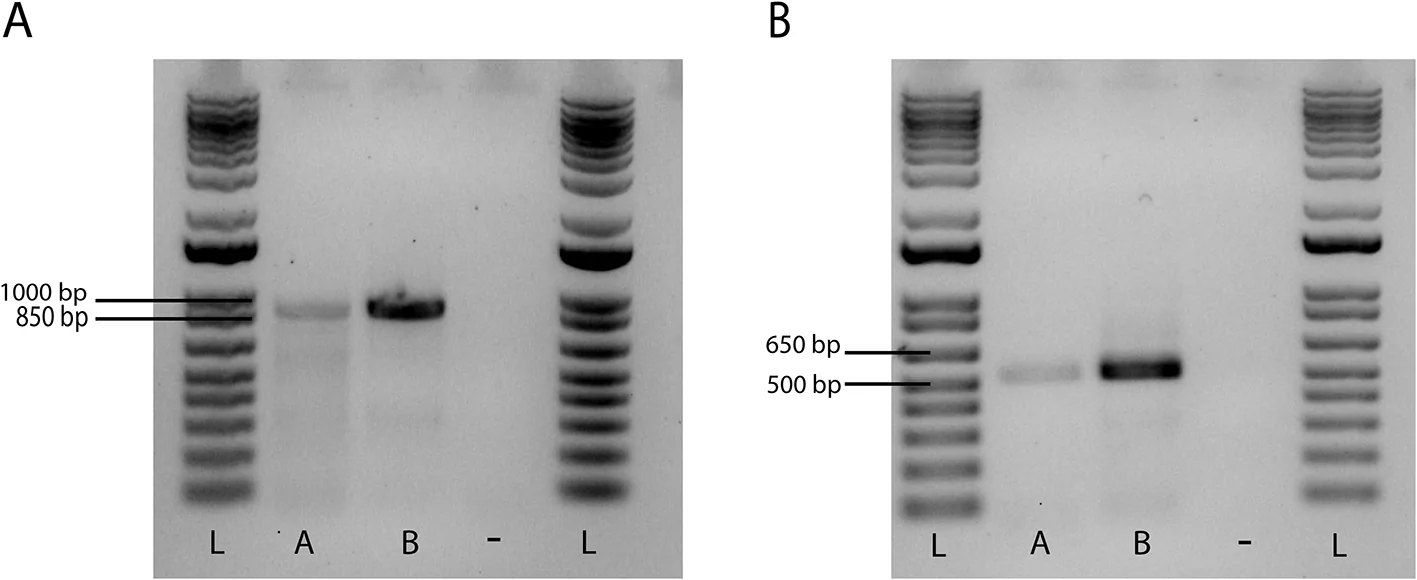
Results from molecular validation of viral presence. **(A)** Shows presence of our proposed new dicistrovirus strain in both years. **(B)** Shows presence of CCYV RNA 1 in both years.

## Discussion

This study identified a small subset of viral targets with strong and repeatable support in field-collected *B. tabaci* samples from Georgia, despite many low-abundance viral taxonomic assignments. CCYV and a dicistrovirus-like sequence related to *Culex dicistrovirus 1* were the only viral targets supported by read assignment, genome assembly, read mapping, and molecular validation in both sampling years. CCYV is a plant-infecting virus transmitted by whiteflies, whereas the dicistrovirus-like sequence is most closely related to insect-associated dicistroviruses. Although whole-insect RNA sequencing does not establish replication, tissue localization, transmission, or reservoir status, the repeated recovery and validation of these two targets distinguishes them from the low-abundance viral assignments and identifies them as the strongest candidates for further biological evaluation.

Mapping trimmed reads to the *B. tabaci* reference genome resulted in approximately 31% high-confidence mapping in both 2023 and 2024. This mapping rate likely reflects the mixed composition of whole-insect RNA, including host, viral, symbiont, and plant-derived sequences, as well as sequence divergence between the Georgia whitefly populations and the reference genome. The reference genome was derived from a geographically distinct population in Spain (BioProject PRJEB47898) but was selected for its chromosome-level assembly, high completeness, and overall sequence quality. Assembly quality was prioritized over exact population correspondence because the reference was used only for host-read removal, not for population-level or comparative genomic analysis. Raw reads from this study were submitted to GenBank under BioProject PRJNA1475529.

COI-based sequence analysis indicated that MEAM1 was the dominant *B. tabaci* signal in both sampling years. Additional non-MEAM1 COI matches were observed, particularly in the 2023 sample, but these matches included shorter or lower-coverage contigs and should be interpreted cautiously. These findings are consistent with reports that MEAM1 is common in the southeastern United States, while MED and other members of the *B. tabaci* complex have also been documented in the region (38). The COI data from this study do not provide strong evidence for an increase in MED abundance between 2023 and 2024. Differences in CCYV read coverage between years may reflect variation in virus titer, sample composition, RNA quality, or sequencing depth, but should not be interpreted as evidence of differences in virus acquisition among *B. tabaci* species. Prior work has shown that MEAM1 and MED can differ in acquisition and transmission of some whitefly-transmitted viruses, including begomoviruses (38). CCYV infection has also been associated with altered biological traits in MED whiteflies (18). Together, the COI and viral read-mapping results support MEAM1-dominated whitefly samples in both years and repeated detection of CCYV in the sampled populations.

Read-mapping results separated the two primary viral targets from the remaining low-abundance viral assignments. CCYV and the dicistrovirus-like sequence related to *Culex dicistrovirus 1* had sufficient read support for genome assembly, read mapping, and molecular validation in both years. In contrast, other viral taxa reported by Kraken2 showed little or no genome coverage when reads were mapped to the corresponding reference sequences. For example, APSE-2 had 131 mapped reads in 2023 and 267 in 2024, Big Sioux River virus had 20 and 29 mapped reads, respectively, and several plant viruses, including pepper chlorotic spot virus and tomato spotted wilt virus, had no mapped reads in either year. Other low-coverage signals included *Choristoneura fumiferana* granulovirus and *Hamiltonella* APSE-1, which were represented by mapped reads but lacked the read support needed for genome recovery or validation. These low-coverage assignments are best interpreted cautiously as incidental viral signals from whole-insect RNA, including plant-derived, symbiont-associated, phage, or feeding-associated RNA, rather than evidence of established infection in *B. tabaci*.

Dicistroviruses are small, non-enveloped positive-sense single-stranded RNA viruses, generally 8–10 kb in length, with a dicistronic genome organization common among insect-associated viruses, including viruses reported from mosquitoes, flies, bees, and aphids (2, 39, 40). The dicistrovirus genome typically contains two open reading frames. ORF1 encodes a nonstructural polyprotein that includes helicase, protease, and RNA-dependent RNA polymerase domains involved in viral replication, whereas ORF2 encodes structural capsid proteins involved in virion assembly and host-cell entry. Some dicistroviruses have been investigated as potential biological control agents, although experimental support varies among viruses and host systems (2, 40). Plant-associated persistence has also been reported for some dicistroviruses, including *Rhopalosiphum padi* virus (RhPV), which has been detected in plant phloem and may facilitate exposure of hemipteran hosts (2, 40). Whether the dicistrovirus-like sequence detected in the present study represents a whitefly infection, plant-associated virus, or insect-to-insect association remains unresolved.

The dicistrovirus-like sequence recovered from the whitefly samples was most closely related to aphid lethal paralysis virus (ALPV) and Culex dicistrovirus 1 strain CDicV1/Fresno. ALPV is a dicistrovirus in the genus *Cripavirus* and has been reported from aphids, where it is associated with paralysis and mortality (41, 42).

Kraken2 assigned reads to ALPV, likely because Culex dicistrovirus 1 was not represented in the viral database used for taxonomic classification. BLASTn and phylogenetic analysis instead placed the recovered sequence closest to Culex dicistrovirus 1 strain CDicV1/Fresno, which was first identified in a metagenomic survey of field-collected *Culex* mosquitoes in California (43). The recovered sequence clustered within a dicistrovirus clade that includes ALPV and Culex dicistrovirus 1, supporting its interpretation as a dicistrovirus-like sequence related to members of the genus *Cripavirus*. However, the available data do not establish infection of whiteflies, host-range expansion, or formal strain status. A contig representing this dicistrovirus-like sequence was assembled, annotated, and submitted to GenBank under accession number PZ562424.

ALPV has been evaluated experimentally for aphid control, and virus-derived genes have also been discussed as potential tools for developing aphid resistance in plants (41, 44, 45). Other dicistroviruses have been investigated or proposed as potential biological control agents against agricultural pests, including moths, aphids, and fire ants (39, 46, 47). However, the dicistrovirus-like sequence recovered in this study should not be interpreted as a candidate biocontrol agent without experimental evidence of infection, replication, pathogenicity, and transmission in whiteflies or other hosts.

Cucurbit chlorotic yellows virus (CCYV) was expected in the whitefly samples because previous studies reported CCYV in cucurbit production systems in Georgia and documented whitefly transmission in the region (16, 16, 48). Little sequence divergence was observed between the 2023 and 2024 CCYV sequences, and phylogenetic analysis did not support classification as a distinct new strain. Instead, the CCYV sequences recovered in this study clustered with previously reported Georgia isolates, including isolate CF-125 (MW685456.1), MW629380.1, and OM489401.1. These results are consistent with prior reports describing CCYV occurrence and additional Georgia isolates after the first report of CCYV in the state (16). Consensus sequences generated from read mapping to CCYV RNA1 and RNA2 were extracted, aligned, annotated, and submitted to GenBank under accession numbers PZ682709 for RNA1 and PZ682710 for RNA2.

Differences in band intensity between the 2023 and 2024 samples likely reflected variation in template quantity or RNA integrity rather than absence of the target sequences. The fainter 2023 bands are consistent with lower RNA input concentrations, which can reduce template availability during reverse transcription and PCR amplification. Longer storage of the 2023 samples before processing may also have contributed to reduced RNA integrity and amplification efficiency. Viral load may also have differed between years, but the qualitative PCR assay does not support direct comparison of viral abundance. Higher read counts in 2024 than in 2023 are consistent with the stronger amplification observed for the 2024 samples (**Tables 2**, **3**).

This study expands current knowledge of viral sequences associated with field-collected *Bemisia tabaci* in the Southeastern United States by recovering near-complete cucurbit chlorotic yellows virus (CCYV) RNA1 and RNA2 sequences and a previously uncharacterized dicistrovirus-like sequence from whitefly samples collected in Georgia. The CCYV sequences clustered with previously reported Georgia isolates and were detected in both sampling years, supporting continued occurrence of this economically important plant virus in the Tifton production region. The dicistrovirus-like sequence was also detected in both years, contained both major open reading frames, and was most closely related to *Culex dicistrovirus 1* and aphid lethal paralysis virus. Although whole-insect RNA sequencing does not establish infection, replication, or transmission, the combined evidence from read mapping, genome assembly, phylogenetic analysis, and molecular validation distinguishes CCYV and the dicistrovirus-like sequence from low-abundance viral assignments. Together, these results provide sequence-resolved evidence for two major viral targets associated with field-collected *B. tabaci* in Georgia and establish a focused basis for future experimental tests of their biological relevance.

## Data Availability

The datasets presented in this study can be found in online repositories. The names of the repository/repositories and accession number(s) can be found in the article/**Supplementary Material**.
